# Ferroptosis: The Silver Lining of Cancer Therapy

**DOI:** 10.3389/fcell.2021.765859

**Published:** 2021-11-29

**Authors:** Zhengming Tang, Zhijie Huang, Yisheng Huang, Yuanxin Chen, Mingshu Huang, Hongyu Liu, Q. Adam Ye, Jianjiang Zhao, Bo Jia

**Affiliations:** ^1^ Department of Oral and Maxillofacial Surgery, Stomatological Hospital, Southern Medical University, Guangzhou, China; ^2^ School of Stomatology and Medicine, Foshan University, Foshan, China; ^3^ Center of Regenerative Medicine, Renmin Hospital of Wuhan University, Wuhan University, Wuhan, China; ^4^ Shenzhen Stomatological Hospital, Southern Medical University, Shenzhen, China

**Keywords:** ferroptosis, cancer therapy, lipid peroxidation, regulatory cell death, reactive oxygen species

## Abstract

Regulatory cell death has been a major focus area of cancer therapy research to improve conventional clinical cancer treatment (e.g. chemotherapy and radiotherapy). Ferroptosis, a novel form of regulated cell death mediated by iron-dependent lipid peroxidation, has been receiving increasing attention since its discovery in 2012. Owing to the highly iron-dependent physiological properties of cancer cells, targeting ferroptosis is a promising approach in cancer therapy. In this review, we summarised the characteristics of ferroptotic cells, associated mechanisms of ferroptosis occurrence and regulation and application of the ferroptotic pathway in cancer therapy, including the use of ferroptosis in combination with other therapeutic modalities. In addition, we presented the challenges of using ferroptosis in cancer therapy and future perspectives that may provide a basis for further research.

## Introduction

**FIGURE 1:**
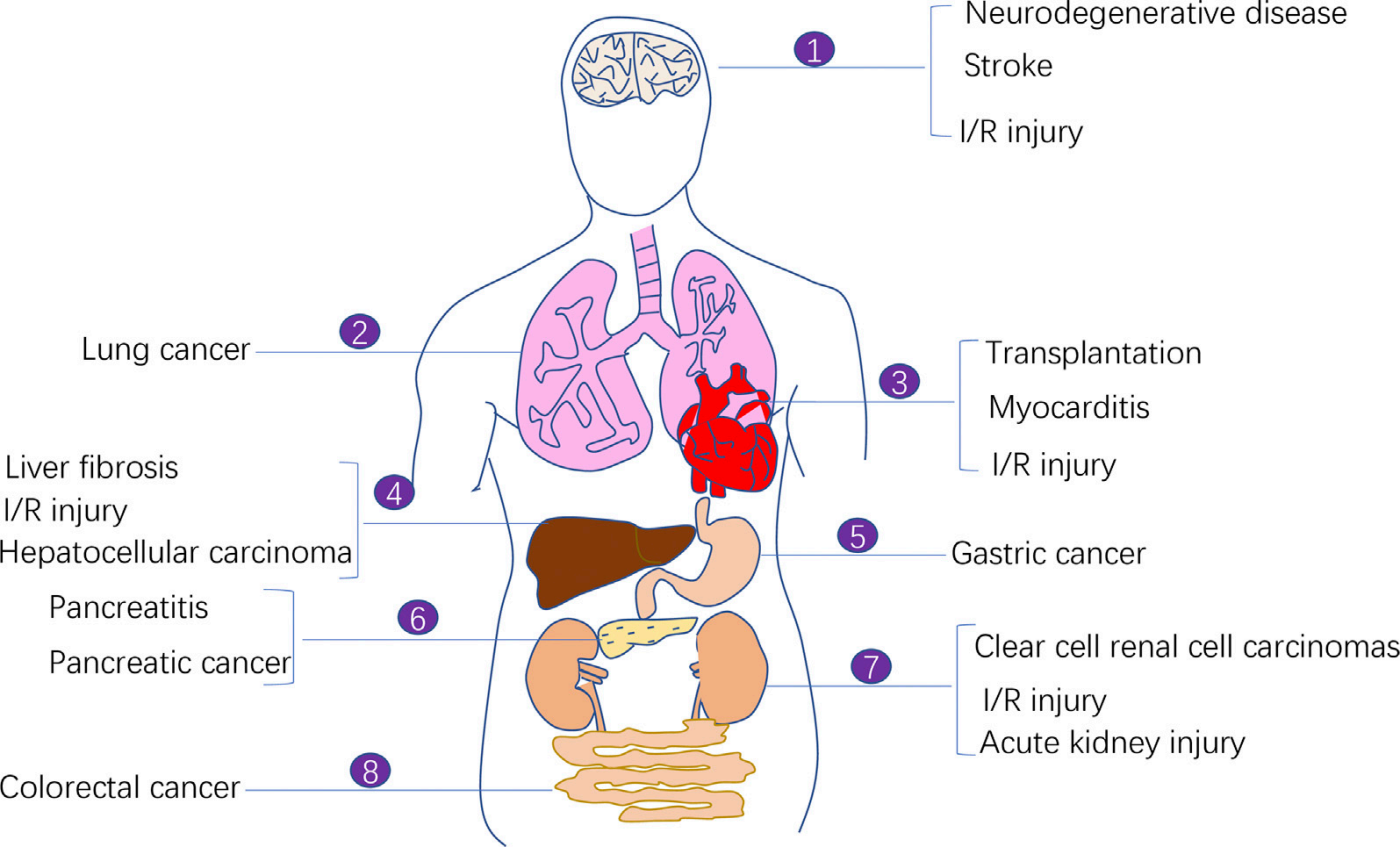
The involvement of ferroptosis in various human diseases. Ferroptosis process is implicated in the pathogenesis of a variety of human diseases including ischemia-reperfusion (I/R) injury diseases, neurodegenerative diseases, stroke, myocarditis, and malignant diseases (e.g., lung cancer, pancreatic cancer, colorectal cancer, clear cell renal cell carcinoma, and gastric cancer).

Cancer is one of the biggest problems plaguing the human life and health. Despite the availability of diverse cancer treatment modalities and the development of modern medicine, cancer therapy faces challenges in selectively killing cancer cells and not damaging normal cells as much as possible to minimise the toxic side effects of treatment. Therefore, targeting regulated cell death (RCD) has become a major focus area of cancer treatment research. A conventional treatment modality was previously used to induce apoptosis in tumour cells using antitumour drugs; however, the therapeutic effect was often limited because tumour cells developed acquired resistance-induced apoptosis (Holohan et al., 2013). Ferroptosis has been increasingly investigated for cancer therapy owing to its specific regulatory mechanism on antitumour-related signalling pathways and its potential target drugs. Increasing studies have demonstrated the therapeutic effects of inducing ferroptosis in cancer cells. In addition to cancer treatment, the ferroptosis process is also associated with many other diseases including ischemia-reperfusion (I/R) injury diseases, neurodegenerative diseases, stroke, myocarditis (Figure 1).

Unlike necrosis and apoptosis, ferroptosis is a form of RCD characterised by iron ion accumulation and substantial lipid peroxidation-mediated membrane damage (Dixon et al., 2012; Stockwell et al., 2017). The concept of ferroptosis was initially proposed in 2012 by Dixon when he used erastin to kill RAS-mutant cancer cells in his study (Dixon et al., 2012). To the best of our knowledge, this is the first review to describe the characteristics of ferroptosis along with its regulatory mechanisms, potential applications in cancer therapy, future perspectives, and challenges.

## Characteristics of Ferroptosis

Ferroptosis is characterised by intracellular iron ion accumulation and reactive oxygen species (ROS)-induced lipid peroxidation (Dixon et al., 2012). As a novel form of cell death, ferroptosis is significantly distinct from other types of cell death in morphology, biological function, genetic mechanism and immunological regulation (Table 1). Ferroptotic cells lead to various cellular morphological alterations, including the loss of cell membrane integrity, partial release of cell contents (e.g. damage-associated molecular patterns [DAMPs]) (Wen et al., 2019), marked reduction in mitochondrial volume, marked loss of mitochondrial cristae and chromatin condensation (Yagoda et al., 2007; Dixon et al., 2012; Friedmann Angeli et al., 2014). Iron chelators (desferrioxamine) have been reported to effectively prevent the occurrence of ferroptosis (Yagoda et al., 2007; Yang and Stockwell, 2008). Furthermore, dysregulation of various genes has been reported to be involved in the biological process of ferroptosis. Various studies have reported the abundant expression of ferroptosis-related genetic biomarkers, including genes related to iron metabolism (e.g. the transferrin receptor [TFR1] and iron-responsive element-binding protein [IREB2]) and lipid synthesis (such as acetyl coenzyme A [CoA] synthase long-chain family member 4 [ACLS4]) (Xie et al., 2016; Mou et al., 2019). In addition, ferroptosis has been found to regulate tumour immunity, and a typical example is HMGB1, a type of DAMP that is released by ferroptotic cells in an autophagy-dependent manner. The released HMGB1 can bind its receptors (e.g. toll-like receptor 4 [TLR4] and advanced glycosylation end-product-specific receptor [AGER]) to mediate the antitumour immune response by stimulating T cells and activating antigen-presenting cells (Wen et al., 2019; Zheng and Conrad, 2020).

**TABLE 1:** Features of different regulated cell death.

RCD	Necroptosis	Apoptosis	Autophagy	Pyroptosis	Ferrroptosis
Morphological Features	Organelle swelling; moderate chromatin condensation	Plasma membrane blebbing; nucler fragmentation	Formation of double-membraned autolysosomes	Cell edema and membrane rupture	Small mitochondria with membrane densities
Biochemical Features	Drop in ATP level; Activation of RIP3	Activation of caspases oligonucleosomal DNA fragmentation	Increased lysosomal activity	Dependent on caspase-1 and proinflammatory cytokine releases	Inhibition of GPX4; Iron ac cumulation; lipid peroxidation
Immune Features	Pro-inflammatory	Anti-inflammatory	Anti-inflammatory	Pro-inflammatoy	Pro-inflammatory
DAMP	DNA and IL6	Ecto-CRT; HMGB1 and ATP	HMGB1	HMGB1 ATP, IL-1β	HMGB1
Regulatory gene	IEF1, RIP1, RIP3	Caspase; P53; Fas; Bcl-2; Bax	ATG5, ATG7, DRAM3, TFEB	IL-18, IL-1β, Caspase-1	GPX4, Nrf2, LSH, TFR1

## Molecular Mechanisms Associated With Ferroptosis

The core molecular mechanism of ferroptosis involves the imbalance between oxidative damage generated by intracellular free radicals and the intracellular antioxidant system (Kuang et al., 2020). Ferroptosis involves oxidative damage to the cell membrane, leading to cell death, and iron ion accumulation and lipid peroxidation are the important markers of oxidative damage.

### Role of Iron in Ferroptosis

**FIGURE 2:**
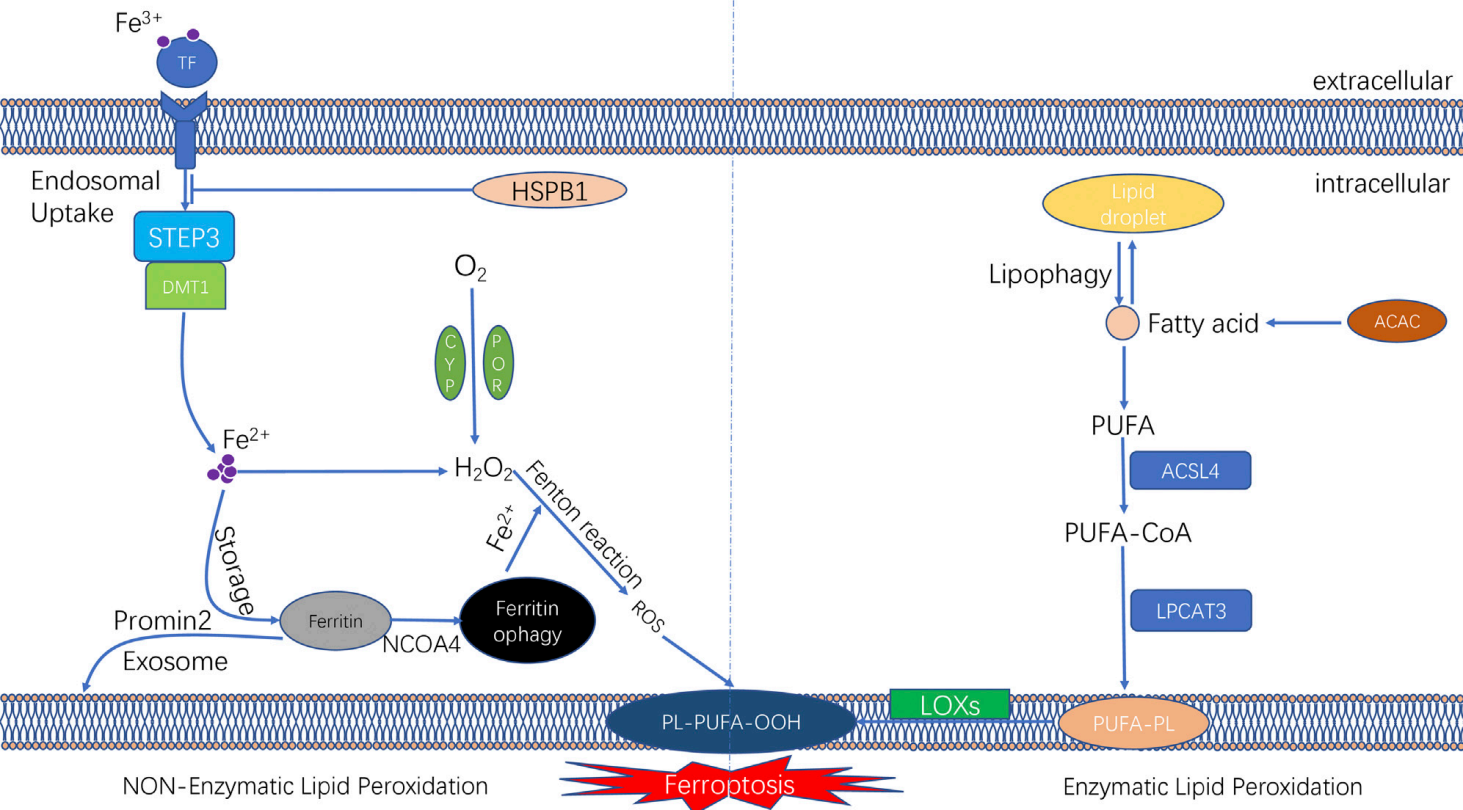
The molecular mechanisms underlying the initiation of ferroptosis. The unique feature of ferroptosis is the iron-dependent lipid peroxide accumulation. The lipid peroxidation can be divided into non-enzymatic lipid peroxidation and enzymatic lipid peroxidation. During the process of non-enzymatic lipid peroxidation, iron ion was transported into cells by transferrinl; the fention reaction between iron ion and H_2_O_2_ lead to the production of lipid ROS. During the process of enzymatic lipid peroxidation, ACSL4 and LPCAT3 promote PUFAs to form PUFA-PLs; and PUFA-PLs undergo lipid peroxidation by lipoxygenases (ALOXs).

Iron is an essential redox-active metal in the cell that is involved in various necessary biological activities. The process of iron ion metabolism in an organism and its role in ferroptosis will be summarised in this section. The trivalent iron ions (Fe3^+^) in the peripheral circulation bind to transferrin (TF) to form a complex and then enter the cellular endosome by binding to the TF receptor (TFR1) on the cell membrane. Simultaneously, Fe3^+^ is reduced to Fe2^+^ (divalent iron ions) by the iron oxygen reductase six-transmembrane epithelial antigen of the prostate (STEAP3). Subsequently, Fe2^+^ is released from the endosome into the cytoplasm, mediated by divalent metal ion transporter protein 1 (DMT1). Some Fe2^+^ released into the cytoplasm is stored in the unstable iron pool (labile iron pool [LIP]). Excess iron is stored as ferritin, and the remaining Fe2^+^ is oxidised to Fe3^+^, which is transported outside the cell by ferroportin (FPN) and participates in iron recirculation *in vivo*. LIP and Fe2^+^ formed by the degradation of ferritin can participate in the intracellular Fenton reaction, which is involved in oxidative stress (Li et al., 2020) (Figure 2).

Iron ions in the intracellular LIP can react with hydrogen peroxide in a Fenton reaction, which lead to the overproduction of reactive oxygen radicals, such as hydroxyl radicals.

The peroxide reaction between hydroxyl radicals and phospholipids containing polyunsaturated fatty acids (PUFAs) occured on the cell membrane can lead to the a series of changes of cell membrane, such as the thinning of cell membrane and formation of protein pores. Such changes of cell membrane affects the balance of the intracellular environment and further causes cell damage (Magtanong et al., 2016). Therefore, changes in the intracellular iron levels through various pathways can impact ferroptosis. Ferritin, another intracellular iron storage, also plays a vital role in the onset of ferroptosis. Nuclear receptor coactivator-4 (NCOA4) mediates the phagocytic degradation of ferritin. NCOA4 overexpression enhances ferritin degradation by binding to ferritin and transporting it from the cytosol to lysosome, leading to increased release of free Fe2^+^ and eventually increased lysosomal ROS production (Torii et al., 2016; Santana-Codina and Mancias, 2018; Battaglia et al., 2020). In addition, indirectly enhancing intracellular ferric ion concentration by increasing the expression of TF and TFR1 can also promote the occurrence of ferroptosis in cells (Shen Y. et al., 2018). However, various interventions that lead to intracellular iron ion depletion can inhibit the occurrence of ferroptosis. Heat shock protein beta-1 (HSPB1) was recently found to inhibit ferroptosis by suppressing the expression of TRF1 to reduce intracellular iron ion concentration (Sun et al., 2015) (Figure 2). A study reported that in iron ion regulation of ferroptosis, intracellular protrusion protein 2 inhibited ferroptosis by promoting iron-containing multivesicular bodies (MVBs) and exosomes to transport iron ions outside the cell, thereby reducing the intracellular iron ion concentration (Brown et al., 2019).

### Lipid Peroxidation and the Antioxidant System

Lipid peroxidation is an essential mechanism of ferroptosis and can be classified as enzymatic and non-enzymatic. The intracellular antioxidant system also plays an essential regulatory role in ferroptosis. In the following sections, we have systematically summarised the involvement of lipid peroxidation and the antioxidant system in the mechanism of ferroptosis (Figure 2).

#### Non-Enzymatic Lipid Peroxidation

Non-Enzymatic lipid peroxidation is a chain reaction driven by free radicals. It is essentially the ROS-induced lipid peroxidation of PUFAs on cell membranes (Hassannia et al., 2019). Intracellular ROS are formed mainly through three modalities: intracellular mitochondrial oxidative phosphorylation; the transmembrane transfer of electrons by nicotinamide adenine dinucleotide phosphate oxidase (NOX); and the Fenton reaction (Kuang et al., 2020). Hydroxyl radicals are one of the most dominant ROS and are mainly derived from the Fenton reaction between iron and hydrogen peroxide.

Recent studies have found that hydrogen peroxide, which undergoes the Fenton reaction, may be associated with intracellular enzymes. Nicotinamide adenine dinucleotide phosphate (NADPH)–cytochrome P450 oxidoreductase (POR) is an oxidoreductase located on the endoplasmic reticulum that transfers electrons from NADPH to microsomal cytochrome P450, cytochrome b5 and haeme oxygenase (HO), thus maintaining the intracellular redox homeostasis (Riddick et al., 2013). NADH–cytochrome b5 reductase 1 (CYB5R1) is another oxidoreductase whose deletion inhibits lipid peroxidation and ferroptosis. It was found that POR bound to CYB5R1 can induce lipid peroxidation in cells. The specific mechanism involves the binding of POR to CYB5R1 to form hydrogen peroxide, followed by the Fenton reaction of hydrogen peroxide with Fe2^+^ to generate hydroxyl radicals (Koppula et al., 2021; Yan et al., 2021).

The first step in non-enzymatic lipid peroxidation is the extraction of hydrogen by hydroxyl radicals from PUFAs in the cell membrane, thereby generating carb-centric lipid radicals (L•). Subsequently, oxygen molecules (O_2_) react with carbon-centred lipid radicals to form lipid peroxide radicals (LOO), which can take up hydrogen from adjacent PUFAs to form lipid hydroperoxide (LOOH) and new lipid radicals to complete the lipid radical chain reaction. In addition, lipid hydroperoxides (LOOH) are converted to alkoxides (LO•) via Fe^2+^, which then react with adjacent PUFAs to induce another lipid radical chain reaction. These processes occur when there is an imbalance between lipid oxidation and the antioxidant system, leading to cell rupture and death and eventually ferroptosis (Hassannia et al., 2019).

#### Enzymatic Lipid Peroxidation

Enzymatic lipid peroxidation is mainly mediated by the lipoxygenase (LOX) family, which is a group of haeme-type iron-containing enzymes that catalyse PUFAs, primarily adrenergic acid (Ada) and arachidonic acid (AA). The first step in enzymatic lipid peroxidation involves that Acetyl-CoA carboxylase (ACAC)-mediated fatty acid synthesis or lipophagy-mediated fatty acid release induces the accumulation of intracellular free fatty acids. The next step in enzymatic lipid peroxidation involves the formation of the corresponding derivatives AA-CoA and Ada-CoA catalysed by ACSL4 (Hassannia et al., 2019). Subsequently, lysophosphatidylcholine group transferase 3 (LPCAT3) catalyses the reaction of AA-CoA and Ada-CoA with phosphatidylethanolamine (PE) to form AA/PE and Ada/PE, respectively (Dixon et al., 2015; Doll et al., 2017). Finally, LOX promotes the peroxidation of AA/PE and Ada/PE to form AA/Ada-PE-OOH, leading to oxidative damage to the cell membrane. Therefore, the ACSL4–LPCAT3–LOX signalling axis is an intracellular pathway that promotes lipid peroxidation in ferroptosis, and inhibition of this pathway may inhibit ferroptosis.

### Antioxidant System

**FIGURE 3:**
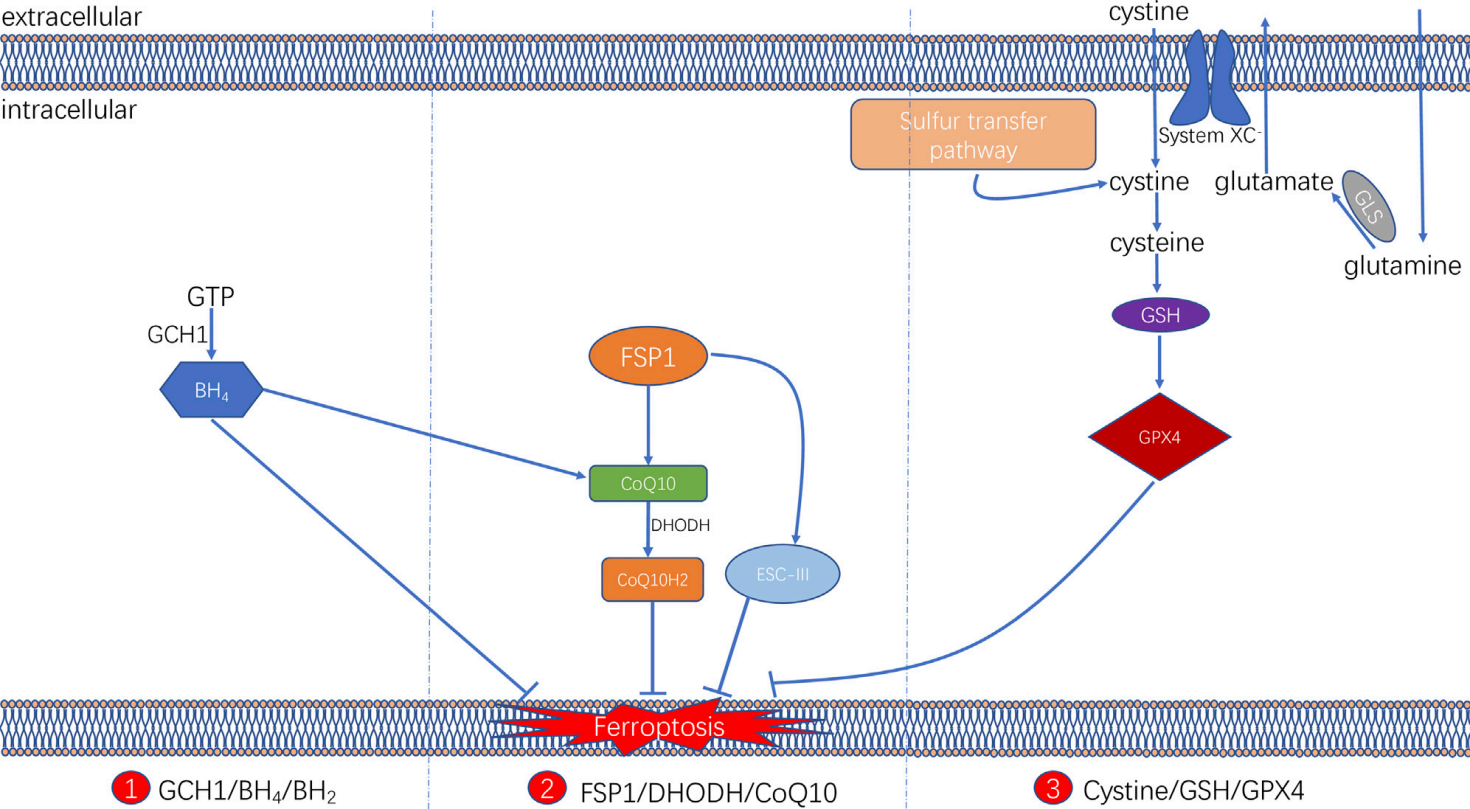
Three signaling axes in the ferroptosis-regulating antioxidant systems, including GTP/GCH1/BH4 axis, FSP1/DHODH/CoQ10 axis, and Cysteine/GSH/GPX4 axis.

It is now known that ferroptosis can be regulated by multiple parallel antioxidant pathways, for example, Cysteine/GSH/GPX4 axis, FSP1/DHODH/CoQ10 axis, and GCH1/BH4/DHFR axis. The implication of these three signaling axes in ferroptosis will be summarized in this section (Figure 3).

#### Cysteine/GSH/GPX4 Axis

The glutathione (GSH) peroxidase (GPX) family is a group of essential enzymes that intracellularly inhibit antioxidants. GPX4 is considered the most important antioxidant enzyme. It uses GSH as a substrate to reduce intracellular peroxides to non-toxic lipid alcohols. Therefore, once GPX activity is inhibited by inhibiting GSH synthesis or by directly inhibiting GPX, inhibition of peroxides can be directly reduced, thus achieving increased intracellular lipid peroxide accumulation and cellular ferroptosis.

GSH is synthesised by cysteine in the presence of r-glutamylcysteine synthetase, and GSH synthetase (GSS). Cysteine synthesis depends on the cystine/glutamate reverse transporter (system XC^–^), which is a membrane Na^+^-dependent amino acid reverse transporter widely distributed in the phospholipid bilayer of biological cells. The transporter system XC^−^ is also a heterodimer composed of a light chain solute carrier family 7 member 11 (SLC7A11) and a heavy solute carrier family three members 2 (SLC3A2). This transporter system excretes a molecule of cystine whenever a molecule of cystine is taken up into the cell (Pitman et al., 2019). Intracellular cystine is reduced to cysteine mediated by thioredoxin reductase 1 (TXNRD1) (Mandal et al., 2010). Followed by this, glutathione (GSH) was synthesized by using cysteine as the substrate. Cysteine–GSH–GPX4 signalling axis is considered the most classical pathway in the regulation of ferroptosis. Inhibiting the system XC^–^ transporter reduces intracellular GSH synthesis and further inhibit GPX4 activity directly, which affects the capacity of the intracellular antioxidant system and thereby leading to the development of ferroptosis. Extracellular glutamate and glutamine levels can affect the occurrence of ferroptosis by affecting system XC^–^. When extracellular glutamate levels are elevated, system XC^–^ can be prevented from performing glutamate/cystine exchange, thereby reducing GSH synthesis and subsequently triggering ferroptosis (Hirschhorn and Stockwell, 2019; Battaglia et al., 2020).

#### FSP1/DHODH/CoQ10 Axis

Two recent studies have identified a regulatory protein similar to GPX4 that is involved in the onset of cellular ferroptosis, namely ferroptosis suppressor protein 1 (FSP1) (Bersuker et al., 2019; Doll et al., 2019). FSP1 was previously known as apoptosis-inducing factor mitochondrial 2 (AIFM2) and has significant homology to apoptosis-inducing factor (AIF) (Wu et al., 2002), a flavoprotein described initially as a P53-responsive gene (Horikoshi et al., 1999) that induces apoptosis.

FSP1 is recruited to the cell membrane via myristoylation and can subsequently reduce ubiquinone (i.e. coenzyme Q [CoQ10]) to ubiquinol in the form of an oxidoreductase. By such reaction, FSP1, acts as a lipophilic radical trap and inhibits cell membrane lipid peroxidation (Bersuker et al., 2019; Doll et al., 2019). In addition, FSP1, as an AIF family protein, can catalyse the regeneration of CoQ10 in reaction with NAD(P)H [31]. In some cases, FSP1 can also inhibit ferroptosis in a pathway parallel to that of CoQ10, such as through the membrane repair mechanism of ESCRT-III (a protein complex). However, the exact mechanism remains unknown (Dai et al., 2020). The FSP1 inhibitor iFSP1, which induced ferroptosis in GPX4-knockdown cells and FSP1-overexpressing cells, was found to exert the intracellular antioxidant effect of FSP1 (Doll et al., 2019).

In addition, a recent study revealed that an antioxidant system might exist in cellular mitochondria (Mao et al., 2021). Dihydroorotate dehydrogenase (DHODH) is located in the inner mitochondrial membrane and is responsible for catalysing the fourth step of pyrimidine nucleotide synthesis, that is, the oxidation of dihydroorotate (DHO) to orotate (OA) and the reduction of CoQ in the inner membrane to ubiquinol (CoH_2_) by receiving electrons. CoH_2_ can act as a trapping antioxidant to prevent intracellular lipid peroxidation (Vasan et al., 2020).

The DHODH inhibitor brequinar was found to induce ferroptosis in GPX4-low cells and enhance the susceptibility of GPX4-high cells to ferroptosis. The results were further validated in an *in vivo* assay via tumour formation in a nude mouse. The assay identified an alternative oxidative pathway to GPX4 in the cytosol and mitochondria and FSP1 in the cell membrane. DHODH in the inner mitochondrial membrane inhibits lipid peroxidation in the mitochondria by reducing CoQ_10_ to CoQ_10_H_2_, indicating that DHODH can be a novel therapeutic target in cancer therapy by inducing ferroptosis.

#### GCH1/BH4/DHFR Axis

In addition, a study demonstrated that a CRISPR/dCas9 overexpression screening using a genome-wide activation library identified the genes signature that inhibit ferroptosis, among which GTP cyclic hydrolase 1 (GCH1) and its metabolic derivative tetrahydrobiopterin/dihydrobiopterin (BH_4_/BH_2_) are shown to be the most prominent (Kraft et al., 2020). In cells, GTP can be hydrolysed by GCH1 to generate BH_4_ and mediates its role in cellular lipid remodelling, thus acting as an antioxidant. It was further found that, unlike the antioxidant mechanism of GPX4, the antioxidant effect of GCH1–BH_4_/BH2 did not protect all PUFA-containing phosphates (PUFA-PLs) from oxidation; instead, it can selectively protect specific phosphates containing two PUFA acyl chains from oxidative degradation, thereby inhibiting ferroptosis. In addition, under oxidative stress following ferroptosis induction, BH_4_ inhibited ferroptosis by increasing CoQ10 levels. Therefore, the implication of GCH1–BH_4_/BH_2_ axis in inhibiting ferroptosis can be either by directly playing the intracellular antioxidant role in protecting cell membrane phospholipids, or by promoting CoQ10 synthesis and further regulating oxidative stress levels. The combination of these two regulating approaches can together form an antioxidant system independent of Cysteine/GSH/GPX4 axis (Kraft et al., 2020).

### Role of Tumour Suppressor Genes in Ferroptosis

Previous investigation have reported that two tumour suppressor genes [p53 and BRCA1-associated protein 1 (BAP1)] played significant roles in regulating tumour by targeting ferroptosis. For example, p53 is an important tumour suppressor gene that regulates cellular responses such as transient cell cycle arrest and apoptosis (Bieging et al., 2014). P53 inactivation plays a crucial role in the initiation and progression of several tumours (Berkers et al., 2013). It has been found that p53 sensitises cells to ferroptosis by inhibiting the expression of SLC7A11, a vital component of system XC^–^, which in turn inhibits cellular uptake of cystine and reduces intracellular GSH synthesis (Jiang et al., 2015). In addition, p53 can reduce GSH depletion and lipid ROS aggregation through the P53–P21 axis, thus inhibiting lipid peroxidation and delaying the onset of ferroptosis caused by cystine deficiency (Tarangelo et al., 2018). Therefore, p53 gene seems to play dual regulating role in ferroptosis, depending on different circumstances. Another tumour suppressor gene-BAP1 can reduce GSH synthesis and induce ferroptosis by suppressing the level of H2A-K119ub on the SLC7A11 gene and the transcription of its mRNA, thereby inhibiting the ability of SLC7A11 to transport cysteine into the cell (Zhang et al., 2018).

### Role of Ferroptosis in Cancer Therapy

Owing to the rapid growth and proliferation of cancer cells, they are more iron-dependent as compared with normal cells. TFR1 upregulation and FPN downregulation have been reported in various cancer cell lines (Gao et al., 2015; Torti and Torti, 2019); therefore, ferroptosis-related genetic biomarkers may be considered potential therapeutic biomarkers for cancer treatment. Ferroptosis-related anticancer drugs and ferroptosis inhibitors are also widely studied in the field of cancer treatment (Table 2).

**TABLE 2:** Ferroptosis inhibitors and mode of action.

Ferroptosis inhibitors	Mechanism
Alpha-tocopherol, Vitamin E, trolox	Block LOX PUFA oxygenation
CoQ10, idenbenone	Target lipid peroxyl radicals
Deferoxamine, deferiprone, ciclopirox	Deplete intracellular iron
Cycloheximide	Block system XC^−^ protein synthesis
Ferrotatins, liproxstatins	Inhibit lipid peroxidation
Butylated hydroxytolune butylated hydroxyanisole	Inhibit lipid peroxidation
Dihydrobiopterin (BH2)	Antioxiant effect
Tetrahydrobiopterin (BH4)	—
Glutaminolysis inhibitor	Hinder mitochondrial TCA cycle
Deuterated PUFAs, MUFAs	Inhibit lipid peroxidation

### Anti-Tumour Drugs Associated With Ferroptosis

**FIGURE 4:**
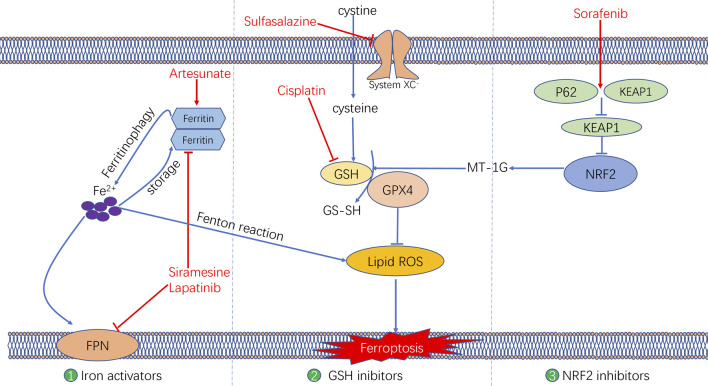
The molecular mechanisms underlying the ferroptosis targeting role of three types of anti-tumor drugs (i.e., Iron activators, NRF2 inhibitors, as well as System XC^−^ and GSH inhibitors).

Various mechanisms can induce cellular ferroptosis, for instance, inhibition of system XC^–^ transport and GPX4 activity or GPX4 degradation. Different antitumour drugs associated with ferroptosis have been developed, including iron activators, NRF2 inhibitors and GSH inhibitors (Su et al., 2020). These drugs have been demonstrated to inhibit tumour growth and exert antitumour effects by inducing ferroptosis. The currently developed ferroptosis-related drug agents are summarised in this section (Figure 4) (Table 3).

**TABLE 3:** Anti-tumor drugs associated with ferroptosis.

Agent	Targets	Possible mechanisms of ferroptosis	Cancer type
Iron activators			
Artesunate	Iron	Promotes ferritin degradation	pancreatic ductal adenocarcinoma
Ferumoxytol	Iron	Iron overproduction	Leukemia
Siramesine	Iron	Inhibit ferroportin and ferritin	Breast cancer
Lapatinib	Iron	Promote the expression of transferrin	Breast cancer
Salinomycin	Iron	Promote the expression of IREB2 and TFRC	Tumour stem cell
Neratinib	Iron	increase the level of iron	Breast cancer, CRC
NRF2 inhibitors			
Sorafenib	SlC7A11, NRF2	activates NRF2 and inhibit system XC^−^	Hepatocellular Carcinoma
Fenugreek	NRF2	inhibit NRF2	head and neck cancer
System XC^-^and GSH inhibitors			
Sulfasalazine	System XC^−^	inhibit the production of GSH	small-cell lung cancer; Prostate Cancer
Cisplatin	GSH	Decrese the level of GSH	Ovarian cancer; pancreatic cancer
Lanperisome	System XC^−^	Inhibit the absorption of cystine	Kras-mutant tumor

### Iron Activators

Artesunate, a semisynthetic derivative of the artemisinin group of drugs, has been commonly used as a frontline drug for treating malaria. In addition, it was also reported to exhibit great potential for cancer treatment. Previous studies have found that artemisinin and its derivatives can exert antitumour effects by inducing cell cycle arrest (Willoughby et al., 2009; Chen et al., 2010), promoting cell invasion and facilitating tumour angiogenesis and metastasis (Zhang et al., 2017). The signalling pathways that mediate the antitumour effects of artemisinin were identified to be the mitochondrial and classic MAPK pathways. A recent study demonstrated that in addition to the conventional mechanism of inducing antitumour effects, artesunate influenced the phenotype of mutationally active KRAS pancreatic ductal adenocarcinoma (PDAC) cells by inducing ferroptosis (Eling et al., 2015). Artesunate induces ferroptosis in tumour cells by enhancing lysosomal activity and increasing lysosomal iron concentration (Yang et al., 2014). A previous study investigated the antitumour role of artesunate by inducing ferroptosis and found that artesunate can alter the mRNA expression level of iron-related genes in tumour cells and further induce cell death in an iron-dependent manner (Ooko et al., 2015). Artesunate regulates ferroptosis by promoting ferritinophagy by regulating the gene expression of NCOA4, which leads to an increase in the iron levels (Kong et al., 2019). The overproduction of ROS triggered by the Fenton reaction between iron ion and hydrogen peroxide is a crucial factor for inducing ferroptosis. Several clinical trials have shown the therapeutic efficacy of targeting ferroptosis in cancer therapy. In a clinical trial regarding metastatic uveal melanoma, patients treated with artesunate had higher survival rates than those of patients in the control group, suggesting that using artesunate to target ferroptosis was beneficial for the prognosis of patients with cancer (Berger et al., 2005). Although several studies have demonstrated the anti-tumour effects of artesunate in cancer treatment, its underlying genetic mechanisms warrant investigation using advanced gene sequencing technology.

Furthermore, two other iron activators are siramesine, which has been commonly used for treating depression, and lapatinib, which is a tyrosine kinase inhibitor. Recent studies have found that siramesine can cause cell death by rupturing cellular lysosomal membranes and can subsequently increase intracellular ROS production. Lapatinib is a potent inhibitor of the epidermal growth factor receptor (EGFR, ErbB-1) and ErbB-2(Wood et al., 2004) The combination of siramesine and lapatinib exerts antitumour effects by inducing ferroptosis, which can be reversed via iron chelators (Ma et al., 2017). A study reported that the combination of siramesine and lapatinib induced intracellular ROS overproduction and ferroptosis by elevating the intracellular free iron levels in breast cancer cells (MDA-MB-231, MCF-7, ZR-75 and SKBr3). In addition, these drugs induced aberrant expression of ferroptosis-related genes, for instance, FPN downregulation and TF upregulation (Ma et al., 2016). Therefore, the study provided a new idea for treating breast cancer through ferroptosis, providing a basis for subsequent clinical cancer treatment. Although these studies have demonstrated evident antitumour effects of using siramesine and lapatinib in treating cancer cells *in vitro*, the *in vivo* application of these drugs requires further research.

### NRF2 Inhibitors

Sorafenib, as a multi-kinase inhibitor, has been acknowledged as a standard therapeutic drug for treating advanced hepatocellular carcinoma (Wilhelm et al., 2006). The underlying mechanism of the antitumour action of sorafenib involves inhibition of tumour angiogenesis and downregulation of the RAF/MEK/ERK pathway (Su et al., 2020). In addition, sorafenib was also reported to exert antitumour effects in various types of tumour cells by triggering ferroptosis (Wang and Lippard, 2005). Sorafenib was reported to induce ferroptosis by activating two signalling pathways including the GSH and p62–Kelch-like ECH-associated protein 1 (Keap1)–NRF2 pathways. Regarding the GSH signalling pathway, sorafenib was found to inhibit the transport function of system XC^–^, which leads to GSH depletion and decrease in the antioxidant capacity (Dixon et al., 2014). With regard to the p62–Keap1–NRF2 pathway, sorafenib was shown to activate the gene function of NRF2 by inhibiting the degradation of Keap1, which is an important component of the p62–Keap1–NRF2 signalling axis. Activated NRF2 regulates ferroptosis by dysregulating the expression of quinone oxidoreductase 1 (NQO1), HO1 and ferritin heavy chain 1 (FTH1). As the first approved antitumour drug targeting ferroptosis, sorafenib faces major challenges regarding drug resistance that should be overcome before its safe application in clinical treatment. An important target causing sorafenib resistance is metallothionein (MT), which is a class of high-level small-molecule reactive proteins that can effectively scavenge ROS (Datta et al., 2007). After being treated by sorafenib, MT1G expression was reported to be upregulated, which inhibited ferroptosis and led to drug resistance by inhibiting the GPX4 gene expression. Therefore, MT1G knockdown via RNA interference technology may be a potential strategy to avoid drug resistance caused by sorafenib in cancer treatment (Sun et al., 2016). In addition, studies have also reported that sorafenib can work synergistically with the ferroptosis inducer erastin in drug-resistant tumour cells, suggesting that the combined use of sorafenib and erastin provides a novel therapeutic strategy for cancer treatment (Lu et al., 2017).

### GSH Inhibitors

The anti-tumour effects of two GSH inhibitors (sulfasalazine and cisplatin) that target ferroptosis will be described in this section. Sulfasalazine is an oral anti-inflammatory drug widely used in clinical practice for treating rheumatoid arthritis or ulcerative colitis (Fleig et al., 1988; Combe et al., 2009). As an inhibitor of the antiporter system XC^–^, sulfasalazine induces ferroptosis in tumour cells (e.g. lymphoma) by suppressing the intracellular antioxidant capacity (Gout et al., 2001). The application of sulfasalazine drug was shown to enhance the treatment efficacy of gamma radiation therapy in glioma treatment, indicating its role in promoting the treatment sensitivity of tumour cells (Sleire et al., 2015). In studies regarding breast and lung cancers, sulfasalazine was found to inhibit drug resistance and reduce side effects of chemotherapy when used in combination with conventional chemotherapeutic drugs (Lay et al., 2007; Narang et al., 2007; Guan et al., 2009). Although various studies have reported the beneficial treatment effects of sulfasalazine in many cancers, whether its anticancer effects are mediated by targeting ferroptosis remains unclear.

Another GSH inhibitor—cisplatin—is a key chemotherapeutic drug agent that has been widely used in the clinical treatment of various solid tumours. It exerts its anti-tumour effects mainly through DNA damage in the cell nucleus, followed by apoptosis (Wang and Lippard, 2005). However, recent studies have reported a novel regulatory mechanism of cisplatin in cancer treatment by demonstrating its ferroptosis-inducing role via GSH depletion and its promoting role in ferritin autophagy (Guo et al., 2018; Zhang et al., 2020). Owing to DNA damage and apoptosis, the anticancer effect of cisplatin often results in drug resistance. Compared with apoptosis, ferroptosis-mediated cell death may not easily lead to drug resistance because ferroptosis as a form of RCD is independent of apoptosis. Therefore, ferroptosis may be a promising pathway to overcome cisplatin resistance. In addition, a recent study demonstrated that cisplatin can synergistically act with the ferroptosis inducer erastin, and the combined use of both drugs was found to enhance antitumour effects when compared with the use of either drug alone (Guo et al., 2018).

#### Gene Technology-Induced Ferroptosis

Genes are essential in life processes and can encode proteins involved in various cellular activities. Some genes have recently been found to play a crucial role in the occurrence of ferroptosis. Therefore, gene technology targeting ferroptosis can be used to treat cancer. The primary gene technologies include gene transfection and gene knockout.The P53 pathway, a classical tumour suppression pathway, has recently been found to sensitise cells to ferroptosis by inhibiting SLC7A11 (Berkers et al., 2013), a positive regulatory gene of ferroptosis. However, ACSL4 is an essential pro-ferroptosis gene (Shen Z. et al., 2018), and ACSL4-mediated production of 5-hydroxyeicosatetraenoic acid (5-HETE) contributes to ferroptosis and is significantly downregulated in ferroptosis-resistant cells, thus positively regulating ferroptosis (Shen Z. et al., 2018). Therefore, by transfecting target cells with ferroptosis-positive regulatory genes such as P53 and ACSL4, ferroptosis can be induced for cancer treatment (Shen Z. et al., 2018). HSPB1 is a negative regulator of ferroptosis. HSPB1 knockdown in cells enhances erastin-induced ferroptosis, whereas HSPB1 overexpression inhibits erastin-induced death (Sun et al., 2015). Ferroptosis-negative regulatory genes can be knocked down in tumour cells using RNA interference technology, which induces cellular ferroptosis and increases anticancer activity. Therefore, gene technology (e.g. gene transfection or gene knockout) can be used in cancer therapy to introduce a relevant regulatory gene into a cell or to silence a gene, thereby regulating cellular ferroptosis (Shen Z. et al., 2018).

### Ferroptosis in Combination With Other Treatments

Despite the efficacy of several clinical modalities for cancer treatment, increasing studies have shown that a single tumour treatment modality is often limited by multiple factors in clinical practice, resulting in unsatisfactory treatment outcomes. However, a combination of various modalities may offer advantages (Lang et al., 2019). Studies have demonstrated that ferroptosis can be associated with multiple cancer treatment modalities, such as radiotherapy and immunotherapy, with promising results (Lang et al., 2019). Therefore, combining ferroptosis with other cancer treatment modalities is also a major focus area for cancer therapy research.

#### Ferroptosis in Combination With Immunotherapy

**FIGURE 5:**
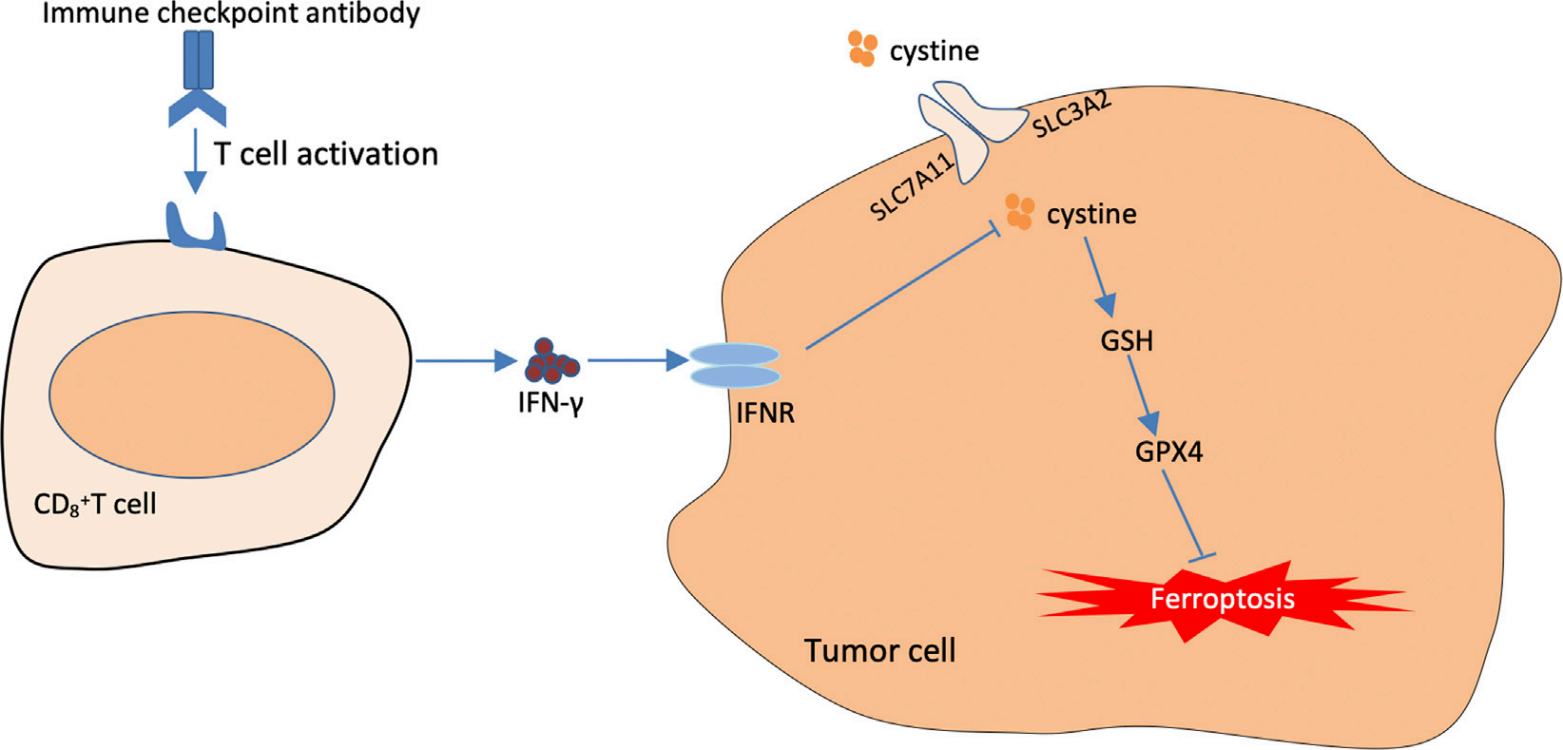
The Mechanisms underlying the promoting role of immunotherapy in targeting cellular ferroptosis. T cells activated by immunotherapy treatment induce ferroptosis by secreting interferon-γ (IFN-γ). IFN-γ can further inhibit cystine uptake by tumor cells, thus further weakening the capacity of intracellular GPX4.

The traditional treatment modalities often fail to achieve good results in clinical practice owing to various drawbacks. Immunotherapy has gradually become an essential strategy for tumour treatment, in which immune-blocking agents exert their antitumour effects mainly by promoting the clearance function of cytotoxic T cells and preventing the escape of tumour cells (Khalil et al., 2016). Cancer immunotherapy is effective in treating some solid tumours including breast cancer, lung cancer, gastric carcinoma. However, compared with other conventional treatment modalities, immunotherapy is less effective in treating some tumours (Pham et al., 2018). Therefore, it is necessary to combine immunotherapy with other treatment modalities to improve its therapeutic effects (Adams et al., 2019). Recent studies have revealed a close relationship between activated CD8^+^ T cells in tumour immunotherapy and ferroptosis. The underlying mechanism involves the downregulation of SLC3A2 and SLC7A11, the two subunits system XC^–^, by interferon-gamma released from immunotherapy-activated CD8^+^ T cells, thus reducing intracellular cysteine synthesis, decreasing antioxidant capacity and promoting lipid peroxidation and ferroptosis in tumour cells (Wang et al., 2019). In addition, it was found in an *in vivo* study in mice that when an immunosuppressant was combined with cysteine proteases (which degrade intracellular cysteine and cystine), they induced ferroptosis in tumour cells and simultaneously enhanced the efficacy of the immunosuppressant, achieving a synergistic effect (Wang et al., 2019) (Figure 5).

Ferroptosis interacts with the immune system through various mechanisms. In addition to the activation of CD8^+^ T cells by immunotherapy to promote ferroptosis, it has been demonstrated that ferroptosis is a form of immunogenic cell death (ICD) (Efimova et al., 2020). Therefore, ferroptotic cells can release a range of DAMPS, including high-mobility group protein B1 (HMGB1) and calreticulin (CRT), which may interact with pattern recognition receptors (PRRs) and phagocytic receptors on immune cells to activate the immune response and adaptive immune system (Shi et al., 2021). Therefore, it is of great clinical significance to study the immunogenicity of ferroptotic cancer cells and investigate their therapeutic effects.

Furthermore, the excellent biological properties of nanomaterials can be used to improve therapeutic effects by combining ferroptosis with tumour immunotherapy. In a study, a bionic magnetic cyst was constructed, in which Fe_3_O_4_ magnetic nanoclusters (NCs) as the core and leukocyte membrane as the shell were combined with transforming growth factor-β inhibitor (Ti) and PD-1 antibody (Pa) to promote the synergistic effect of ferroptosis and immunomodulation. Under magnetic resonance imaging (MRI)-guided drug delivery, Pa and Ti in bionic magnetic vesicles created an immunogenic environment after entering the tumour interior, which increased the concentration of hydrogen peroxide in polarised M1 macrophages, promoting a Fenton reaction with iron ions released from NCs. Hydroxyl radicals generated from the Fenton reaction subsequently induced ferroptosis in tumour cells, and the tumour antigens released by the dead cells subsequently promoted ferroptosis. The tumour antigens released by the dead cells, in turn, enhanced the immunogenicity of the microenvironment. Therefore, immunotherapy and ferroptosis exert a powerful synergistic effect (Zhang et al., 2019).

Although the relationship between ferroptosis and immunotherapy is not well understood, preliminary studies have been conducted on immunotherapy and ferroptosis in animal models. A study developed an iron oxide-loaded nanovaccine (IONV) for combined ferroptosis–immunotherapy treatment (Ruiz-de-Angulo et al., 2020). The principle of this nanovaccine was based on chemically integrated iron catalysts and drug delivery systems by exploiting the properties of the tumour microenvironment. IONV activation in the tumour microenvironment increased oxidative stress in tumour cells, which in turn promoted ferritin deposition, activated M1 macrophages, improved tumour antigen cross-presentation and enhanced the immunotherapeutic efficacy. The findings from the above-mentioned studies suggest that the combination of ferroptosis and immunotherapy is a promising cancer treatment strategy, which requires in-depth research.

#### Combined Application of Ferroptosis and Chemotherapy

Chemotherapy is a common clinical approach to cancer treatment, and the commonly used chemical agents include methotrexate, cyclophosphamide and vincristine. Although chemotherapy has made significant progress in clinical cancer treatment, the epithelial-to-mesenchymal transition, in which tumour cells can metastasise and become resistant to drugs, has limited the effectiveness of anticancer therapies (van Staalduinen et al., 2018; Yang et al., 2020). Epithelial-to-mesenchymal transition is a process wherein epithelial cells lose polarity and intercellular adhesion and gradually transform into invasive and metastatic mesenchymal cells (Yang et al., 2020). However, it has been found that mesenchymal cells formed after epithelial-to-mesenchymal transition have a higher susceptibility to ferroptosis (Hangauer et al., 2017; Viswanathan et al., 2017). Therefore, the use of ferroptosis appears to be one of the strategies to address tumour drug resistance and metastasis (Xu et al., 2021).

Inducing ferroptosis in drug-resistant tumour cells can overcome drug resistance, and varied underlying mechanisms have been proposed. Ferroptosis inducers can reverse tumour drug resistance by promoting intracellular ROS accumulation, thereby inducing ferroptosis in drug-resistant tumour cells (Friedmann Angeli et al., 2019). Furthermore, NRF2 can reduce intracellular ROS accumulation via the P62–Keap1–NRF2 pathway, which in turn is involved in the resistance of cancer cells to sorafenib; however, NRF2 inhibitors can reverse the resistance of cancer cells by increasing intracellular ROS accumulation (Roh et al., 2017).

Furthermore, ferroptosis inducers can induce ferroptosis in drug-resistant tumour cells by decreasing cellular TF levels and increasing intracellular iron transfer. For example, artesunate can induce ferroptosis in Adriamycin-resistant leukaemia cells by decreasing TF levels and promoting the therapeutic effect of Adriamycin on leukaemia (Wang et al., 2017; Park et al., 2018).

Ferroptosis agonists that act by inhibiting the system XC^–^ transport function can also lead to lipid peroxidation in resistant cells, thereby reversing drug resistance in tumour cells (Lim et al., 2005). For example, erastin can reverse cisplatin resistance in ovarian cancer cells by inhibiting the transport function of system XC^–^ (Sato et al., 2018).

In addition to reversing tumour cell resistance, the use of ferroptosis agonists in combination with chemotherapeutic agents for non-drug-resistant cancer cells can also achieve more desirable results. Ferroptosis can adopt the mechanism of action of traditional chemotherapeutic drugs, such as cisplatin, which function by reducing intracellular GSH by inhibiting GPX4 activity to induce cellular ferroptosis (Guo et al., 2018). Therefore, a combination of the chemotherapeutic drug cisplatin and ferroptosis inducers may enhance therapeutic efficacy. For example, erastin combined with cisplatin provides better results than when cisplatin is used alone (Guo et al., 2018). Another ferroptosis inducer, RSL3, exerts its induction effect by inhibiting GPX4 when combined with cisplatin, which enhances the therapeutic influence of cisplatin both *in vitro* and *in vivo* (Zhang et al., 2020). In addition to cisplatin, other classical chemotherapeutic agents can also exert better effects when used along with ferroptosis inducers. For example, the classical chemotherapeutic drug paclitaxel can further regulate glutamate catabolism (one of the pathways through which ferroptosis occurs) in colorectal cancer cells by reducing the expression of intracellular glutamate-catabolism-related genes such as SLC7A11 and SLC1A5 and inducing an increased expression of P53 and P21, which altogether inhibit tumour growth (Lv et al., 2017). In mutant-P53 pharyngeal squamous carcinoma, when low doses of paclitaxel were combined with the ferroptosis inducer RSL3, paclitaxel upregulated mutant P53 and promoted RLS3-induced ferroptosis, providing a synergistic therapeutic effect (Ye et al., 2019).

Therefore, the emergence of ferroptosis combined with chemotherapy as a new treatment modality offers a new strategy for tumour treatment.

#### Combination of Ferroptosis and Radiotherapy

Radiotherapy is one of the therapeutic modalities for the clinical treatment of tumours, which involves direct and indirect mechanisms of action. Regarding its direct mechanism, high-energy ionising radiation disrupts the double-stranded structure of cellular DNA and leads to cell cycle arrest. Regarding its indirect mechanism, radiation leads to cellular alterations including radiolysis of intracellular water and stimulation of oxidative enzymes. These alterations can generate ROS such as hydroxyl radicals and hydrogen peroxide. ROS overproduction can further damage nucleic acids, proteins and lipids, thereby inducing cell death (Lei et al., 2020). A recent study demonstrated that radiotherapy can exert therapeutic effects by inducing ferroptosis in cells, and the combination of radiotherapy and ferroptosis inducers results in enhanced antitumour effects when compared with the use of either treatment alone. The results of the study indicated that the combination of ferroptosis and radiotherapy might provide a promising strategy for treating tumours (Lang et al., 2019).

**FIGURE 6:**
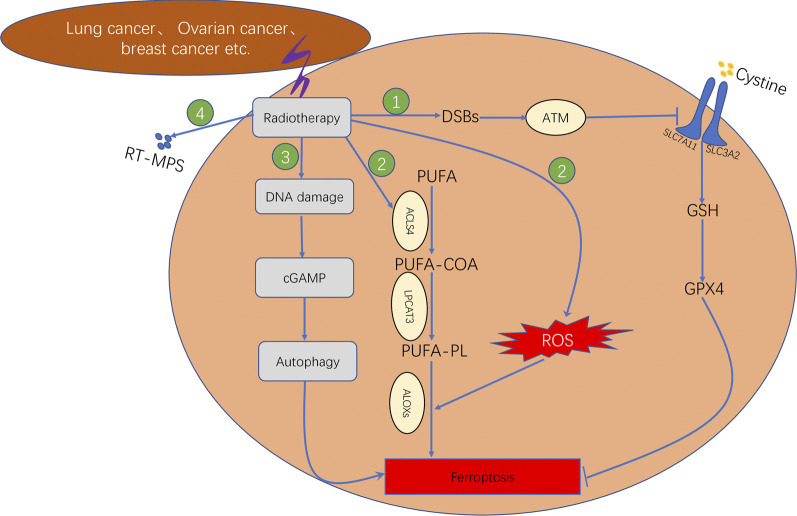
Mechanism underlying ferroptosis induced by radiotherapy, which consisted of four steps. First step: Radiotherapy affects GSH synthesis by affecting system XC^−^ transport via ATM. Second step: Radiotherapy promotes lipid biosynthesis by upregulating ACSL4 expression. Third step: Radiotherapy induces autophagy-dependent ferroptosis by causing DNA damage. Fourth step: Radiotherapy facilitates the production of RT-MPs; thereby causing lipid peroxidation in neighbouring cells.

In addition, recent studies have reported that radiotherapy can induce ferroptosis in tumour cells. The four primary underlying mechanisms can be summarised as follows (Figure 6: First, radiotherapy can upregulate the expression ofataxia–telangiectasia mutated (ATM) gene and further inhibit the transport function of system XC–through inducing DNA double strand breaks (DBSs), which leads to the inhibition of antioxidant activity of GSH and GPX4, therebyresulting in lipid peroxidation (Lang et al., 2019). Second, radiotherapy can either increase the production of ROS or upregulate the gene expression of ACSL4. The conversion of PUFA to PUFA-CoA is catalysed by ACSL4. Subsequently, the PUFA-CoA enzyme is converted to PUFA-PL, which is catalysed by LPCAT3. The PUFA-PL product can induce lipid peroxidation owing to the catalysis of arachidonate lipoxygenases (ALOXs). Therefore, radiotherapy can promote intracellular lipid peroxidation and induce ferroptosis (Lei et al., 2020; Lei et al., 2021). Third, tumour cells treated with radiation can release tumour cell-released microparticles (RT-MPs) into the extracellular environment, which can promote the effect of radiotherapy by inducing ferroptosis in other tumour cells (Wan et al., 2020). Fourth, radiotherapy induces intracellular DNA damage, thereby activating the CGAS–cGAMP signalling pathway and subsequently inducing autophagy-dependent ferroptosis (Li et al., 2021).

Regarding the application of ferroptosis in radiotherapy, the combination of radiotherapy and the ferroptosis inducer (FIN) was found to act synergistically in tumour treatment by increasing lipid peroxidation and upregulating PTGS2 expression, with FINS exerting significant radio-sensitising effects *in vitro* and *in vivo* (Lei et al., 2020). Another study reported similar results by demonstrating that the ferroptosis inducer sulfasalazine acted as a radiosensitiser and increased the sensitivity of gliomas to Gamma Knife radiosurgery. The underlying mechanism involves blocking the uptake of cystine by system XC^–^, leading to GSH depletion (Sleire et al., 2015). Another study investigating lung adenocarcinoma and glioma established a murine xenograft model and human patient-derived models to analyse the beneficial effects of combining radiotherapy with the ferroptosis inducer sorafenib *in vivo*, resulting in the improved efficacy of radiotherapy and inhibition of tumour growth (Ye et al., 2020).

Furthermore, a study reported that ferroptosis was also involved in radiotherapy-induced cellular damage in normal cells instead of only affecting tumour cells (Li et al., 2019). Based on this study, the combined application of ferroptosis inducers and radiotherapy in cancer treatment needs to be considered with caution. Whether the combination causes less harm to normal tissues than to tumour tissues needs to be investigated. Further studies to address this issue are warranted, and nanomaterials may be a future research direction. Altogether, these studies demonstrate that the combination of radiotherapy and ferroptosis may be considered a novel therapeutic strategy and also provides directions for future cancer research.

#### Combined Application of Ferroptosis and Photodynamic Therapy

Photodynamic therapy (PDT) is a non-invasive cancer treatment modality. The action mechanism of PDT involves absorbing energy from ionising radiation and generating cytotoxic singlet oxygen (Dolmans et al., 2003). However, the efficacy of PDT in tumour treatment is limited owing to the inherent hypoxic nature of the tumour microenvironment. Therefore, oxygen supplementation in the tumour microenvironment is an essential measure to improve the efficacy of PDT (Cui et al., 2018). Because ferroptosis can generate lipid ROS, it is an excellent strategy to synergise with PDT.

Some studies have reported the applications of ferroptosis-related drugs in PDT therapy. A previous study designed a co-assembled nanosystem using the combination of erastin (ferroptosis inducer) and chlorin e6 (photosensitiser Ce6). This nanosystem was formed via intermolecular interactions of hydrogen bonding and π–π stacking. Erastin increased the concentration of intracellular oxygen and significantly improved the therapeutic efficiency of PDT by generating ROS (Zhu et al., 2019). Another example of the combined use of oxygen-enhanced PDT and ferroptosis is a 2-in-1 nanoplatform (SRF@HB-Ce6), which was designed by piggybacking the photosensitiser Ce6 on sorafenib (ferroptosis inducer) attached to the haemoglobin (Hb) (Xu et al., 2020). As for this nanoplatform, the capacity of Hb in providing endogenous iron and carrying oxygen provided the conditions for photodynamic-type therapy by enhancing ferroptosis (Xu et al., 2020). In addition, PDT can enhance ferroptosis and sensitise tumour cells to ferroptosis by recruiting immune cells to secrete interferon-gamma. Such 2-in-1 nanosystems combine the therapeutic advantage of both ferroptosis-related drugs and PDT and play a significant role in promoting antitumour effects (Xu et al., 2020). In addition to these nanosystems established by incorporating specialised photodynamic sensitisers (e.g. ferroptosis inducers), another design method has recently emerged based on the ability of some nanomaterials to generate ROS under certain conditions. For example, graphene oxide was recently found to release ROS under near-infrared (NIR) irradiation (He et al., 2017). Based on this theory, a research group prepared a new graphene oxide-based NIR-absorbing nanoparticle agent that was decorated with iron hydroxide/oxide (GO-FeO xH). *In vitro* experiments on this nanomaterial demonstrated that GO-FeO x H released oxygen upon exposure to NIR, which improved the treatment outcome of PDT (He et al., 2017).

#### Ferroptosis and Nano-Therapy

Owing to the unique physicochemical properties of nanomaterials, bio-nanotechnology has become a focused research topic in the field of cancer treatment. Drug treatment can be improved by delivering anticancer drugs via nano-drug delivery systems (nano-DDS), which can significantly reduce the toxic side effects of drugs.

Recent studies have reported the use of nanomaterials to efficiently deliver ferroptosis targeting drugs (Shen Z. et al., 2018). Because ferroptosis is cell death caused by intracellular iron ion-dependent lipid peroxidation and lipid ROS accumulation, nanomedicines developed for ferroptosis are mainly used for intracellular ROS assembly. Therefore, based on the action mechanism of ferroptosis, ferroptosis-related nanodrugs can be classified into the following three categories: first, ferroptosis-related nanodrugs that can promote intracellular Fenton reaction; second, nanodrugs that can inhibit intracellular GPX4 activity, thus reducing intracellular antioxidant capacity; third, nanodrugs that can be used for exogenous regulation of lipid peroxidation in tumour cells (Shan et al., 2020). The regulatory role and application of these three categories of ferroptosis-related nano-drugs will be discussed in this section.

The first category of ferroptosis-related nanodrugs (for promoting intracellular Fenton reaction): The Fenton reaction is defined as the reaction between intracellular iron ions and hydrogen peroxide, leading to the production of the harmful hydroxyl radicals. Ferroptosis can induce cell death by promoting the intracellular Fenton reaction (Qian et al., 2019). Compared with normal cells, tumour cells possess more hydrogen peroxide and hence served as a substrate for the Fenton reaction, further facilitating the induction of ferroptosis. In addition, the tumour microenvironment is slightly acidic compared with the normal cell environment, and such Fenton reactions generally do not occur in the relatively neutral normal cells/tissues. Therefore, based on the Fenton reaction, ferroptosis-related drugs can accurately target tumour cells instead of destroying normal cells, which is a major advantage, especially involving less toxic side effects.

The Fenton reaction can be induced in tumour cells by enhancing the substrate of intracellular Fenton reaction (e.g. Fe^+^ and H_2_O_2_) via sustained release by a nano-delivery system. A typical example is nanocatalysts, which are prepared by adding ultrafine Fe_3_O_4_ nanoparticles and natural glucose oxidase (GOD) to silica nanoparticles with large pore size. GOD can effectively consume glucose in tumour cells, thus generating a large amount of hydrogen peroxide. Furthermore, Fe_3_O_4_ reacts with hydrogen peroxide, leading to a Fenton reaction, followed by the overproduction of hydroxyl radicals, which increases ROS levels and further induces ferroptosis in cells (Huo et al., 2017).

The second category of ferroptosis-related nanodrugs (for inhibiting intracellular GPX4 activity): In addition to promoting the intracellular Fenton reaction, these nanodrugs can inhibit GPX4 activity, thus reducing intracellular GSH levels. Currently, there are two primary modes of combining inhibition of GPX4 activity with nanomaterials: mode 1, intracellular administration of GPX4 inhibitors via nano-delivery systems; mode 2, direct GPX4 inhibition using nanomaterials or nanodrugs (Shan et al., 2020).

Mode 1 (direct administration of GPX4 inhibitors via nano-delivery systems): In a study, a nanomaterial scaffold was incorporated with three components (small molecule GPX4 inhibitor sorafenib, Fe^3+^ and tannic acid). Such nanodrugs can not only inhibit GPX4 activity but also convert Fe^3+^ to Fe^2+^ via tannic acid. These alterations eventually induce ferroptosis in cancer cells (Liu et al., 2018).

Mode 2 (nanosystems with GPX4 inhibition): This mode involves designing nanomaterials or drugs with GPX4 inhibition. A typical example is low-density lipoprotein (LDL) nanoparticles reconstituted with the natural omega-3 fatty acid docosahexaenoic acid (LDL-DHA), which were reported to induce ferroptosis in hepatocellular carcinoma cells by inhibiting intracellular GPX4 expression and reducing GSH levels (Ou et al., 2017). Another example is arginine-rich manganese silicate nanobubbles (AMSNs), which were found to induce iron sagging by inactivating GPX4, leading to an efficient GSH depletion capacity. The surface coating of arginine and the nanobubble structure improved the GSH depletion efficiency of manganese silicate nanobubbles when compared with the conventional nanoparticles (Wang et al., 2018).

The third category of ferroptosis-related nanodrugs (for regulating exogenous lipid peroxidation): Ferroptosis is mainly caused by lipid peroxidation, and the primary substrates of lipid peroxidation are PUFAs present in the phospholipid membranes. Therefore, increasing PUFA concentration exogenously may be a reasonable strategy for regulating lipid peroxidation and effectively inducing ferroptosis. This drug design strategy can efficiently improve the antitumour activity of ferroptosis inducers. To verify this hypothesis, some studies have been recently conducted using nano-delivery systems to exogenously modulate lipid peroxidation. A typical example is the nanosystem design based on Fenton-like reactions wherein linoleic acid hydroperoxide (LAHP) was incorporated into iron oxide nanoparticles (IONPs) using sub-stable FeO and Fe_3_O_4_ as iron sources to achieve on-demand Fe^2+^ release from the nanosystem under acidic conditions in tumours. The released Fe^2+^ generated tumor-specific ^1^O_2_ using LAHP, ultimately inducing ferroptosis in tumour cells (Zhou et al., 2017).

## Future Perspectives and Challenges

**FIGURE 7:**
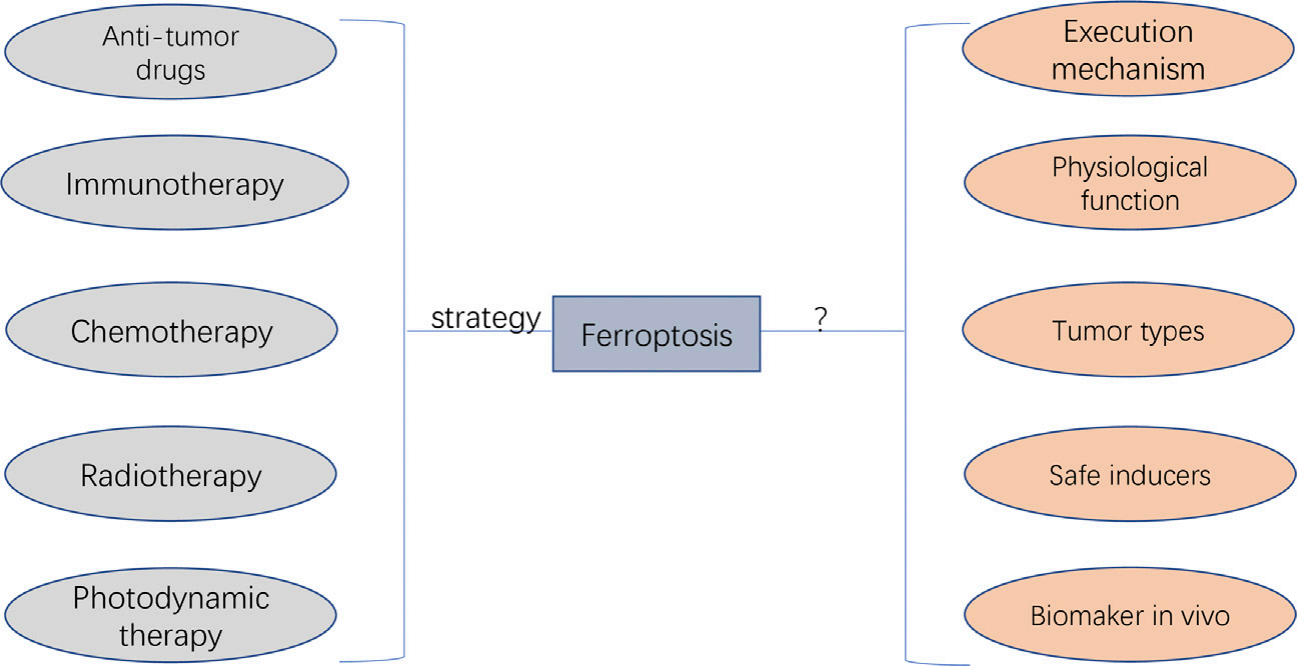
The potential application and faced challenges of ferroptosis in clinical cancer therapy. The anti-tumour drug agents targeting ferroptosis can be developed, which can be applied by the combination of other therapeutic approaches (i.e., immunotherapy, chemotherapy, radiotherapy, and photodynamic therapy). The unaddressed concerns before clinical transfer include unknown execution mechanism, unexplored physiological function, unexpected regulating roles depending on cancer types, undiscovered ferrpotosis inducers applied safely in human, and undetected specific biomarkers for diagnosing the initiation of ferropotosis *in vivo*.

Ferroptosis was initially discovered as a novel form of RCD in 2012, and its underlying transduction mechanisms have recently gained increasing attention in cancer research (Hassannia et al., 2019; Mou et al., 2019). Based on the currently known pathways of ferroptosis (e.g. system XC^–^, GPX4, FSP1 and BH1), various ferroptosis inducers and techniques have been discovered and potentially applied in cancer treatment (Liang et al., 2019). In addition to the sole application of ferroptosis in treating cancer cells, ferroptosis can also be combined with other conventional therapeutic modalities, such as the combination of ferroptosis inducers and chemotherapeutic drugs based on nano-DDS. Such combinational modalities may significantly improve therapeutic efficacy as compared with the effects of either treatment alone and may be a promising strategy for cancer treatment (Shan et al., 2020) (Figure 7).

Despite the leaps and bounds in ferroptosis-based cancer treatment, it still faces several challenges, especially in terms of regulatory mechanisms and its potential application in clinical cancer therapy (Figure 7). The action mechanism of iron-dependent cell death remains unclear and requires extensive investigation. Other regulated cell deaths are executed mainly through proteins and proteases (such as caspases). Whether ferroptosis is caused by the products of lipid peroxidation or by the products downstream of lipid peroxidation remains unclear. In addition, there are insufficient studies regarding the physiological function of ferroptosis during growth and development. Therefore, understanding the role of ferroptosis in growth and development is important for using ferroptosis for further development of cancer therapy.

In addition, there are some unaddressed issues regarding the potential application of ferroptosis in cancer therapy. The first unsolved research question is whether the specific ferroptosis inhibitors and inducers can be applied *in vivo* more safely. Sorafenib, sulfasalazine, statins and artemisinin mentioned in previous sections are currently being investigated as ferroptosis inducers; however, their application in clinical practice requires further investigation. In addition, controlling the toxic effects of ferroptosis inducers on normal tissues is a major concern. Another problem is the selection of tumour types or patients more likely to be treated with ferroptosis-related regimens (Chen et al., 2021). Different tumour types and patients may have different sensitivity to ferroptosis owing to differences in iron levels and expression levels of ferroptosis-related genes. Therefore, iron levels, gene expression and mutations can be used as criteria for judging populations more likely to benefit from ferroptosis-promoting therapies. In addition, ferroptosis-inducing agents need to be significantly improved, especially regarding their pharmacokinetic and physicochemical properties. Currently used ferroptosis inducers have several physical and chemical drawbacks that prevent their rational use in clinical practice. Nanosystems can be improved to some extent in this regard. However, when combining ferroptosis with nanosystem therapies, it should be noted that outcome differences may exist between animals and humans because ferroptosis is non-apoptotic death, which should be further validated. Furthermore, effective combinational strategies that can synergistically work with ferroptosis induction in cancer therapy may provide an experimental direction for future research. Although the combination of ferroptosis with other treatments has been shown to improve the clinical outcomes of cancers, an optimal combination strategy involving ferroptosis and other treatments has not yet been identified. Moreover, there exists neither an accurate method nor an indicator for detecting ferroptosis. Various features of ferroptosis have been identified; however, a specific biomarker of ferroptosis has not yet been identified. At the level of lipid peroxidation, the BODIPY 581/591 C11 fluorescent probe can be used to detect the degree of intracellular lipid peroxidation and hence ferroptosis. At the iron level, ferroptosis can be detected indirectly by using an iron kit to detect the intracellular iron concentration. At the genetic level, PTGS2, ACSL4, CHAC1 and TFRC170 are considered ferroptosis-associated genetic biomarkers based on preclinical models. Although these genes are well known to be significantly involved in ferroptosis, further studies should focus on discovering more specific biomarkers and detection tools for ferroptosis, which will be beneficial for providing meaningful guidance to clinical practitioners.

## Conclusion

Ferroptosis is a promising strategy for cancer therapy; however, further research is required for evaluating its clinical applications.
